# *Ex vivo* evaluation of antibiotic sensitivity in samples from endodontic infections

**DOI:** 10.1080/20002297.2022.2160536

**Published:** 2022-12-22

**Authors:** Álvaro Villanueva-Castellote, Carmen Llena Puy, Miguel Carda-Diéguez, Álex Mira, María D. Ferrer

**Affiliations:** a Department of Stomatology, Universitat de València, Valencia, Spain; b Genomics&Health Department, Foundation for the Promotion of Health and Biomedical Research of Valencia Region (FISABIO), Valencia, Spain; c CIBER in Epidemiology and Public Health (CIBERESP), Instituto de Salud Carlos III, Madrid, Spain

**Keywords:** Antibiotic, biofilm, biofilm model, endodontic, real time cell analysis

## Abstract

**Objective:**

To develop an in vitro model for real-time monitoring of endodontic biofilm growth and evaluate the ex vivo effect of antibiotics on biofilm growth.

**Material and Methods:**

Root canal samples were taken from 40 patients and inoculated into 96-well plates in a system that measures biofilm growth through electrical impedance. Biofilm bacterial composition at the genus and species level was analyzed by Illumina sequencing. ANCOM-BC corrected data were used to compare bacterial composition after antibiotic treatment through compositional analysis, and to compare microbiological with clinical data.

**Results:**

The stationary phase was reached at 8 hours. The biofilm formed had a similar bacterial composition to the inoculum, and Enterococcus faecalis was virtually absent from the samples. The bacterial composition and the effect of antibiotics were sample-dependent. Metronidazole was the antibiotic that most inhibited biofilm formation and azithromycin the one that inhibited it in the highest percentage of cases. The antibiotic effect could not be related to the biofilm original bacterial composition.

**Conclusions:**

The impedance system allowed real-time monitoring of endodontic biofilm formation, and we propose it as a model for ex vivo evaluation of the whole biofilm susceptibility to antimicrobials, as opposed to evaluating antibiotic sensitivity of specific bacterial isolates.

## Introduction

The complexity of the root canal system, together with the polymicrobial nature of endodontic infections, makes disinfection an extremely complex challenge [1,2]. Primary or secondary root canal infections and apical periodontitis (extra-radicular) are mediated by biofilms [3–5]. Growth in the form of biofilms provides micro-organisms with a number of advantages and abilities that are not observed for individual cells living in a planktonic state. This enhances their growth, and may increase diversity, virulence and metabolic efficiency, as well as protect against various factors such as other competing microorganisms, host defenses, antimicrobials and environmental stress [6].

The use of endodontic biofilm study models has as its main objectives the study of its composition, the analysis of microbial interactions and the evaluation of the efficacy of different root canal disinfection procedures [7,8]. There are different models for the study of endodontic biofilms; however, there is currently no ideal standardized system [8]. Some are single-species models, the most predominant being *Enterococcus faecalis* as it has been considered the main causative agent of persistent root canal infections [9]. Some systems, referred to as defined engineering models, are also used where well-characterized model species are combined [10]; these species are composed from 2 [11] to 9 different species [12]. Finally, in some studies, a natural biofilm or microcosm is used [13–15], consisting of samples directly obtained from the environment of interest. This results in a biofilm that is much more complex and closer to reality, but with an initially unknown composition [10]. Among the study models that use a natural biofilm, few allow the study of the complete biofilm dynamics in all its growth phases and in real time [15], as most of them are limited to quantifying the amount of biofilm at the end of the test, losing information on the rest of the stages.

The xCELLigence® Real Time Cell Analysis (RTCA) system is an equipment that allows continuous quantification of biofilm formation using electrical impedance values. This model consists of a microtitre plate with gold microelectrodes at the bottom of the wells through which the current flows. The presence of adhered cells prevents the electrical current flow, whereas planktonic growth does not affect it. In addition, the magnitude of this impedance (similar to electrical resistance) correlates with the cell number and with biofilm mass [16].

Impedance systems have been shown to allow the growth of both single-species and multi-species biofilms, consisting of hundreds of different microorganisms, such as those from saliva, supra- and subgingival plaque or tongue samples [15]. Furthermore, the bacterial composition of the biofilm formed is similar to that of the inoculum from which they originate, and it has been shown that the system can assess the *ex vivo* antibiotic susceptibility of the entire biofilm as a whole [15]. However, this system has not been evaluated for endodontic biofilms.

The correct management of an endodontic infection consists of debridement of the infected root canal together with drainage of the soft and hard tissues [17]. In certain situations where there is systemic involvement or the extent of the infectious process is progressive, antibiotic treatment is necessary [17,18]. Microorganisms present in a biofilm are up to 100 times more resistant to antibiotics than in a planktonic form [19]. Thus, it is of vital importance to know the behaviour of endodontic biofilms against different antibiotics.

Therefore, the objectives of the present study were to develop and test a fast RTCA system as an *in vitro* model to study the growth of endodontic biofilms from root canal samples, as well as to evaluate the *ex vivo* effect of some of the most commonly used antibiotics in the clinical setting -such as amoxicillin, metronidazole and azithromycin- on the formation and bacterial composition of these biofilms.

## Materials and methods

### Donor selection

The present study was approved by the Ethics and Human Research Committee of the Universitat de València (UV), with registration number H148473676706. Inclusion criteria were over 18 years of age, one or more teeth that responded negatively to cold sensitivity tests (negative vitality was confirmed by the absence of bleeding after opening), no antibiotics in the last month, no communication between the buccal environment and the pulp chamber. Teeth with periodontal pocket >4 mm, mobility or root fracture were excluded.

### Sample collection

**Figure 1.**
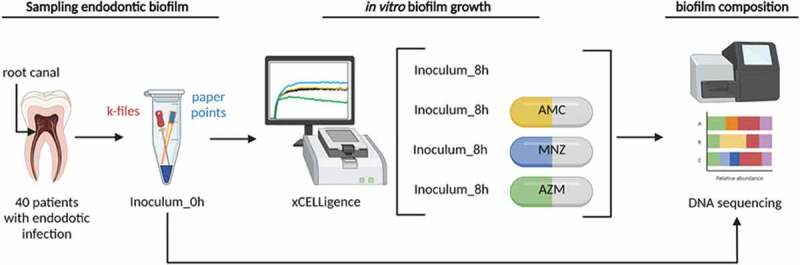
Workflow for microbial analysis of endodontic infections. A schematic view of sample collection, processing, growth and analysis is shown for the antibiotic susceptibility tests, performed with the conditions optimized in the initial set up experiments (1:2 sample dilution and growth for a period of 8 hours). In short, samples from the inside of the necrotic teeth canals were obtained, and the files and paper points with the sample were placed in an Eppendorf tube with PBS. An aliquot was taken from each sample and used for the study of microbial composition by 16S rRNA gene Illumina sequencing. Samples were also inoculated into the wells of E-plates from a xCelligence® RTCA system and incubated at 37°C for 8 hours to study the dynamics of endodontic biofilm formation. Each well contains 100 µl of culture medium with each of the three antibiotics tested (AMC- medium with amoxicillin-clavulanic acid, MNZ- medium with metronidazole and AZM- medium with azithromycin), 100 µl of sample cell suspension (inoculum) and 50 µl of sterile mineral oil to facilitate the creation of anaerobic conditions. Once the biofilm reached the stationary phase (8 hours), the biofilm attached to the bottom of the well was collected for DNA extraction and 16S rRNA gene sequencing, which was carried out using Illumina Miseq followed by bioinformatics analysis with compositional analysis.

For root canal sampling, the tooth was isolated with a rubber dam and disinfected with 2% chlorhexidine applied by wiping with a sterile cotton swab and manipulated with sterile gripper. The access to the cavity was done with a sterile diamond bur. When the ceiling of the pulp chamber was reached, the bur was replaced with a sterile one cooled with sterile saline. Once it was verified that there was no bleeding and the tooth could be included in the study, 1 ml of sterile saline solution was introduced into the canal. Sample collection from inside the canals was carried out using 8, 10 and 15 sterile k-files together with 6 sterile paper points and sterile saline under aseptic conditions. The files and paper tips were placed in an Eppendorf tube with PBS (phosphate buffered saline) [20,21]. The sample was stored in cold storage (4-10°C) and brought to the laboratory in less than 3 hours, accordingly with Vengerfeldt, et al [22]. This was the time necessary to finish clinical activities and prepare the experiment. A simplified flowchart showing the protocol for sample collection and processing is shown in Figure 1.

### Sample processing and growth conditions of biofilms

The sample was vortexed and centrifuged (2 minutes at 6000 rpm), then the paper points and files were removed, and the supernatant was discarded. The pellet obtained was resuspended in culture medium (referred as ‘Med’ in the figures and labels) to obtain the cell suspension for *in vitro* assays. The culture medium used was BHI (Brain Heart Infusion) supplemented with haemin menadione (5 µL/mL) and vitamin K (10 µL/mL) [23].

To determine the number of conditions that could be tested in the impedance RTCA system for the same sample, a set of pilot experiments were performed where the microbial pellet was resuspended in 400 uL of culture medium (Med) and serial dilutions of the cell suspension were performed. For each cell suspension, the ability to form a biofilm was studied in the *in vitro* model. Real-time biofilm analysis was carried out according to the manufacturer’s instructions [24]. Briefly, the reference background was obtained by placing 100 µL of Med in the wells (96 E-plate wells) following the standard protocol [16]. Next, 100 µL of the cell suspension from each dilution was added, followed by 50 µL of sterile mineral oil (Sigma M8410) to promote anaerobic growth conditions. Each sample was run in duplicate with the corresponding negative control medium. The E-plate was then placed in the xCELLigence® RTCA system, incubated at 37°C, and impedance measurements were registered to provide Cell Index (CI) values (equivalent to biofilm growth mass) every 10 min for 24 h. Biofilm growth results for each sample were shown as the mean of the two replicates. The optimal growth time to reach stationary phase (8 hours) and the maximum dilution of the sample in which biofilm growth was observed was taken as a reference for the second phase of the work, which aimed at determining antibiotic susceptibility of the biofilms.

### Quantification of the *ex vivo* effect of antibiotics

Culture medium (Med) was prepared with different antibiotics at the following concentrations: amoxicillin + clavulanic acid (8/1.14 µg/mL) (MedAMC), metronidazole (16 µg/mL) (MedMNZ) or azithromycin (0.4 µg/mL) (MedAZM). The concentrations of each antibiotic were selected based on the maximum serum concentration reached for each antibiotic when 875/125 mg of amoxicillin + clavulanic acid, 500 mg of metronidazole or 500 mg of azithromycin are taken orally [25]. Thus, the amount added to the culture is the one expected to reach in the periradicular tissue. Inside the necrotic root canal, if there is a necrosis, the antibiotic will reach with greater difficulty; however, the periapical biofilm in a necrosis is similar to that of the canal (see references 26 and 27) and the arrival of the antibiotic to the periapical tissue is possible. In each well, 100 ul of the medium with the different antibiotics (Med, MedAMC, MedMNZ, MedAZM) were placed and a background impedance measurement was performed. Next, 100 µL of the processed sample cell suspension was added to each of the wells, using the same procedure as described above, followed by 50 µL of sterile mineral oil to promote anaerobic [26] conditions. Each antibiotic test was carried out in duplicate, including their corresponding impedance controls. The E-plate was placed in the xCELLigence® RTCA system and incubated at 37°C for 8 hours.

Once the impedance values were obtained for each of the wells, the replicates were averaged for each condition. In addition, the percentage of inhibition or stimulation of biofilm formation was calculated with respect to the control sample without antibiotics. An [27] antibiotic was considered to produce an effect when the inhibition or stimulation was equal to or greater than 10% of the biofilm mass formed in the non-antibiotic control.

### Identification of bacterial composition by sequencing

Once the biofilm reached the stationary phase (8 hours), the supernatant was discarded and two gentle washes with 150 ul of phosphate buffer were performed to remove non-adhered cells. Subsequently, the biofilm attached to the bottom of the well was collected, following Mira *et al*. 2019 [15]. DNA from the grown biofilms and from the inoculum from which they were derived was extracted with the MagnaPure LC JE379 instrument using the MagnaPure LC DNA Isolation Kit (Roche®) following the manufacturer’s instructions with some modifications [28].

For sequencing, Illumina amplicon libraries were made following the Illumina protocol for preparation of a 2 × 300 bp library of the 16S rRNA gene (Part #15044223 Rev.A), as described in Dzidic *et al*. 2018 and Mira *et al*. 2019 [15,28]. The quality filtering, chimera removal, end-trimming and analysis of the sequences was performed following the protocol described in Schmieder & Edwards 2011, Mira *et al*. 2019, and Edgar 2016 [15,29,30]. Specifically, the forward and reverse reads were trimmed, removing the v5-v6 primer sequences and low-quality bases at the end of reads, and sequences with any ambiguous N base or exceeding 5 expected errors were discarded. The forward and reverse pairs were merged, with a overlap of 12 bases and a 1 bp maximum mismatch in the overlapping region, to obtain single denoised variants. After chimeric removal, the Silva database [31] (v138) was used as reference for taxonomic assignment at the ASV level using the DADA2 naive Bayesian classifier method [32]. Bacterial abundance at the genus and species levels was normalized using ANCOM-BC, to account for the compositional nature of 16S rRNA sequencing data [33]. All 16S rRNA sequences are publicly available in the SRA database, with BioProject ID PRJNA873285.

### Statistical analysis

To compare the bacterial composition between the different samples before and after the antibiotic treatment, a canonical correspondence analysis (CCA) and an ADONIS test (Permutational Multivariate Analysis of Variance Using Distance Matrices) were performed.

Using the ANCOM-BC approach, the mean abundance of the most abundant genera (those at a proportion >2%) in each sample used as inoculum was compared with the abundance of these genera in the biofilms formed after 8 hours of incubation. In addition, the mean proportion of the most abundant genera (>2%) in the biofilm grown without antibiotic was compared to the bacterial composition after treatment with amoxicillin + clavulanic acid, metronidazole or azithromycin. Wilcoxon test was used to compare diversity indexes (Chao1 and Shannon). ANCOM-BC, CCA and Wilcoxon analyses were performed with R (version 3.6.0), using the Vegan [34] and ade4 [35] packages. P-values were adjusted using the False Discovery Rate method. For these analyses, a species or genus was included if it was present in 70% of the samples from at least one of the two groups with an abundance superior to ten times the smallest percentage above zero. Data were visualized with GraphPad PRISM (v9).

## Results

### Clinical characteristics of the selected patients and samples

Forty-two patients were selected for the study, from which 48 samples were obtained -one per patient, except for six patients from whom samples were obtained from two different teeth-. In 14.6% of the samples obtained (7/48), not enough material was obtained to form biofilms in the xCELLigence® RTCA system. One sample was also discarded due to *Bacillus* contamination. Therefore, the final sample size included 40 samples, 42.5% from men and 57.5% from women. The age of the patients ranged from 18 to 81 years old with a mean age of 44.6 ± 17.1 years.

Of the 40 samples included in the study, 9 were analyzed in the set-up phase to understand the full dynamics of biofilm formation and were grown for 16 h; the remaining 31 were analyzed to determine the effect of antibiotics on biofilm growth for 8 h (the time at observed stationary growth phase). For the analysis of bacterial composition after antibiotic treatment, 6 samples were discarded due to a negligible effect on biofilm formation. Thus, the total number of individuals selected for DNA extraction was 25, from which the following samples were sequenced: i) Inoculum (endodontic root canal sample collected from the patient and stored at −20ºC for sequencing); ii) Biofilm (8 h biofilm formed in the RTCA system); iii) Amoxicillin + clavulanic acid (biofilm treated with amoxicillin + clavulanic acid); iv) Metronidazole (biofilm treated with metronidazole); and v) Azithromycin (biofilm treated with azithromycin).

### Development and validation of the RTCA impedance system for samples of endodontic origin

**Figure 2.**
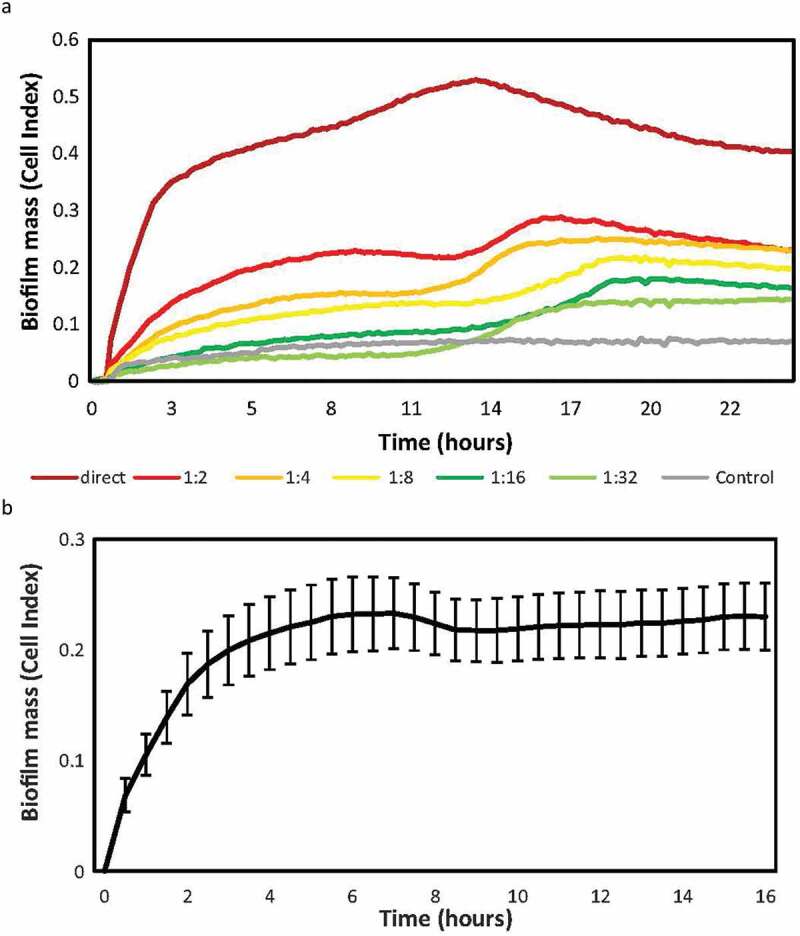
Endodontic biofilm formation dynamics after serial dilutions. **a)** the y-axis indicates the impedance values (i.e. biofilm growth) measured over 24 h. Sample was diluted from an initial concentration 1/1 (pellet diluted in 500 ul of medium), up to a dilution 1/32. The results show the mean Cell Index (CI) or biofilm growth values of two replicates recorded in real time over 24 h of biofilm formation on an RTCA xCelligence system. The grey line corresponds to the negative control of the medium without sample. **b)** Mean and SE (standard error) of endodontic biofilm growth of 9 samples of different patients, measured in real time for 16 h on the RTCA xCelligence® system. For each sample, the mean of the 2 replicates was taken and the impedance values were subtracted from the blank (control medium).

Due to the limited material per sample, the maximum number of conditions or compounds that could be tested for the same sample in the *in vitro* model was first determined. For this purpose, serial dilutions of the sample were performed, and the biofilm-forming capacity of each sample assessed. Figure 2a shows the real-time biofilm formation dynamics of serial dilutions of the same sample measured on the xCELLigence® system, showing a decrease in the biofilm mass obtained as the dilution of the original sample increases. It was therefore established that the maximum dilution of the sample to maintain a Cell Index (i.e. biofilm mass) > 0.2 was 1:2. This implied resuspending the pellet in 1000 µL of culture medium and using 100 µL per well. Thus, the number of possible conditions to be tested was limited to 4, with 2 replicates per condition, including 200 µL for the analysis of bacterial composition in the original sample.

Next, to establish the time at which the biofilm of endodontic samples reaches its maximum growth peak in the system under the described conditions, the biofilm formation dynamics of 9 samples were studied over 16 h of growth (Figure 2b). A first adhesion phase is observed, followed by an exponential growth phase during the first 2 hours, reaching the stationary phase of biofilm growth at 8 hours. Given that the maximum time of endodontic biofilm growth was observed at 8 hours, the rest of samples were grown and analyzed up to that time.

**Figure 3.**
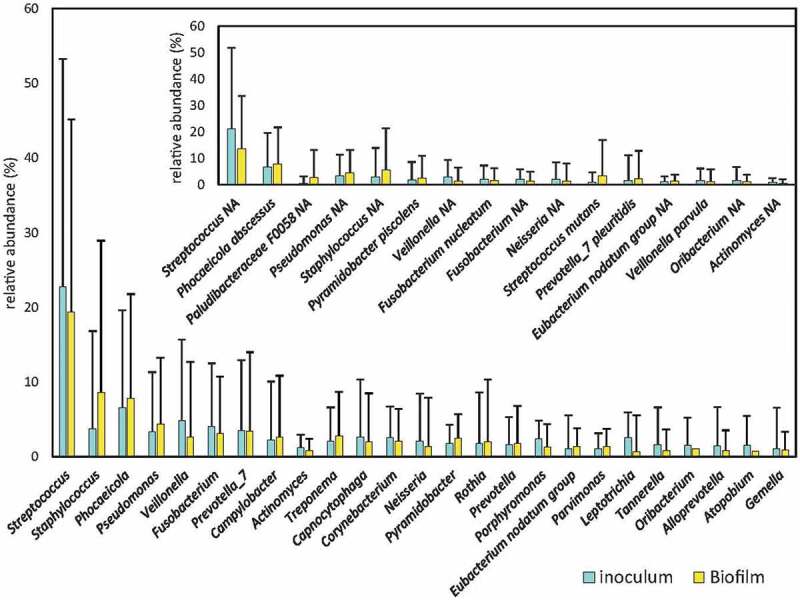
Bacterial composition of root canal infections. Graphs show the mean proportion and SE (standard error) of the most abundant genera in the samples (those at >1% levels) from inside the root canal (inoculum) and the derived biofilms grown *ex vivo*. Inlet graph shows the proportion of the 15 most abundant bacterial species in the samples. * (p < 0.05).

To determine whether the biofilm formed at the bottom of the wells of the xCELLigence® RTCA system was a multi-species biofilm similar in composition to the inoculum from which it was derived, the bacterial composition between the inoculum and the resulting biofilm were compared. Bacterial composition in the endodontic samples was extremely heterogenous among patients (Figure 3). The average proportion of the top-20 bacterial genera detected for the original endodontic infection samples is provided in Supplemental Material 1. The results of the 16S rRNA gene Illumina sequencing analysis showed that there were no statistically significant differences in any bacterial genera present at a proportion higher than 1% (Figure 3), suggesting that the system allows the growth of a biofilm whose bacterial composition is similar and representative of that inside the root canals. Among the species present at more than 1% of abundance in any of the conditions, *Corynebacterium matruchotii*, and unclassified species of *Leptotrichia*, *Fusobacterium* and *Neisseria* had significantly lower abundance after incubation, whereas *Streptococcus mutans* and unclassified species of *Corynebacterium* and *Staphylococcus* underwent an increased abundance after incubation (all ANCOM-BC compositional analysis).

### *Ex vivo* effect of different antibiotics on endodontic biofilm formation

**Figure 4.**
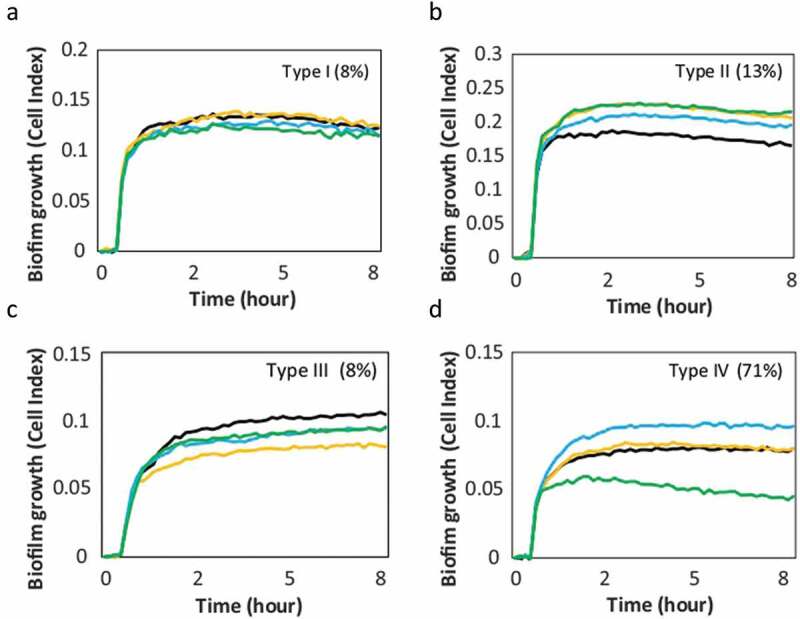
Patterns of *ex vivo* growth of endodontic biofilms derived from necrotic root canals under different antibiotics. The curves show the biofilm growth measured in real time on the xCelligence® RTCA system in the absence of antibiotics (positive control, black curve) or in the presence of antibiotics: amoxicillin + clavulanic acid (yellow), metronidazole (blue), azithromycin (green). Panels a, b, c and d show representative examples of the effect on biofilm formation from each of the 4 patterns found in response to the antibiotics. The percentage frequency of the different biofilm growth patterns found in the 40 samples studied are indicated between brackets. -Type I: no effect; Type II: all antibiotics induce; Type III: all antibiotics inhibit; Type IV: variable effects for different antibiotics-.

After optimizing sample processing and culture conditions, the antibiotic susceptibility tests could be performed for whole endodontic samples. The results of these *ex vivo* antibiotic sensitivity studies on the formation of biofilms from 40 necrotic root canal samples revealed that the effect observed with the RTCA impedance system could be grouped into 4 different response patterns: Type I – No antibiotics affect biofilm growth; Type II – All antibiotics induce more biofilm formation; Type III – All antibiotics inhibit biofilm formation; Type IV (most frequent) - Each antibiotic has a different effect on biofilm growth dynamics. Representative examples of each of the patterns is displayed in Figure 4. An antibiotic was considered to have no effect when the inhibition or stimulation of biofilm was less than 10% compared to the non-antibiotic control [36].

One of the advantages of the real-time continuous monitoring system is that the antibiotic effect can be quantified at different points of biofilm growth. For example, Figure 4 shows that the effect of inhibition or induction remains similar at 4 and 8 h for all biofilm behavior patterns, which may have positive practical implications by decreasing the time needed for antibiotic susceptibility assessment.

**Figure 5.**
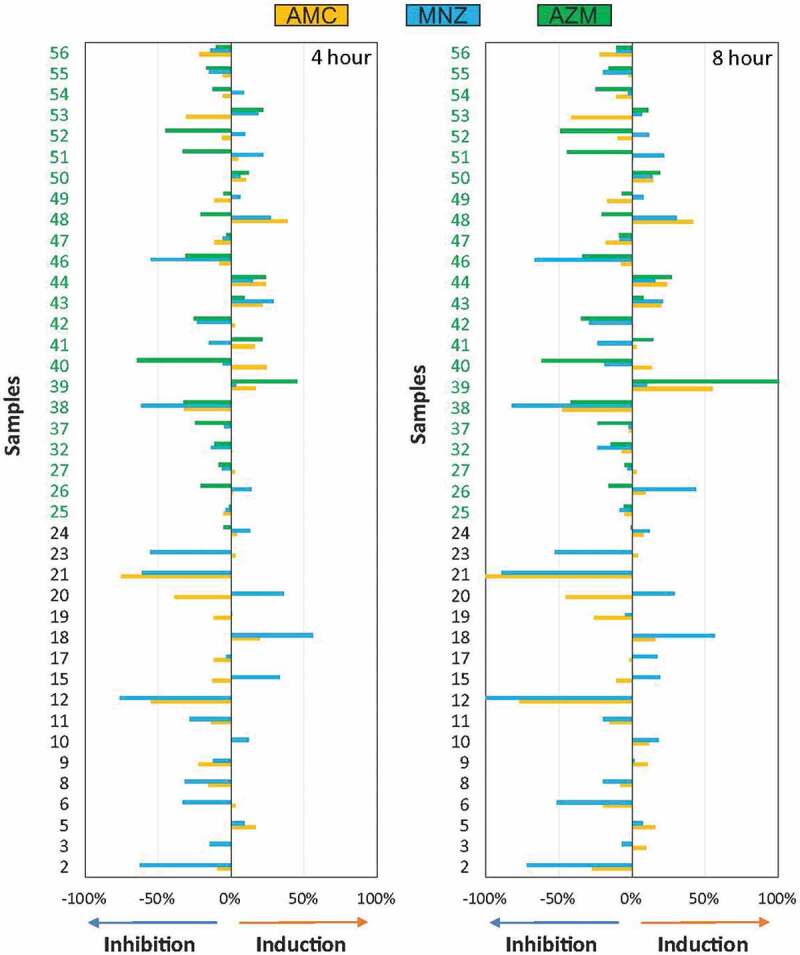
Graphical representation of the percentage of inhibition or induction of biofilm formation *in vitro*. Bar graphs show data from 40 samples of endodontic origin, grown in the presence of different antibiotics, at 4 and 8 hours, compared to a control without antibiotics.

After quantification of the percentage of inhibition or induction of biofilm formation for each of the antibiotics tested, a large variability in the antibiotic efficacy was detected (Figure 5). As observed before, there was virtually no difference in the effect of the different antibiotics on biofilm formation between 4 and 8 hours after antibiotic addition.

**Figure 6.**
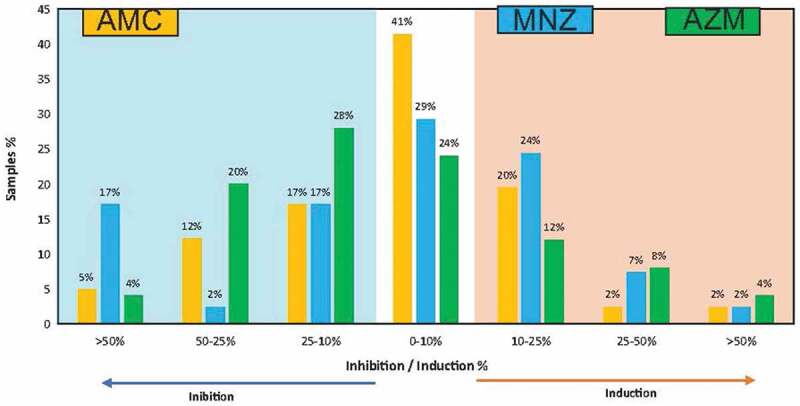
Inhibition or induction frequency of biofilm formation for the different antibiotics tested *ex vivo*. Bar graphs show data from 40 endodontic samples grown for 8 hours. The bars express the percentage of samples in which each antibiotic induced or inhibited biofilm formation with respect to the control sample without antibiotics, for different degrees of efficacy.

In relation to the frequencies of inhibition and induction of biofilm formation associated with each antibiotic and compared to the untreated control (Figure 6), metronidazole was the antibiotic that inhibited biofilm formation to the highest degree (>50% of the biofilm mass) in the highest number of cases (17% of the samples studied), while azithromycin was the antibiotic that had an inhibitory effect between 10–50% in the highest number of samples (48%). In terms of biofilm induction, metronidazole produced a mild induction of biofilm formation (10–25%) in the highest number of cases (24%), followed by amoxicillin + clavulanic acid (20%). Regarding the induction of biofilm formation (>25%), no large differences were observed between the different antibiotics (Figures 5 and 6).

**Figure 7.**
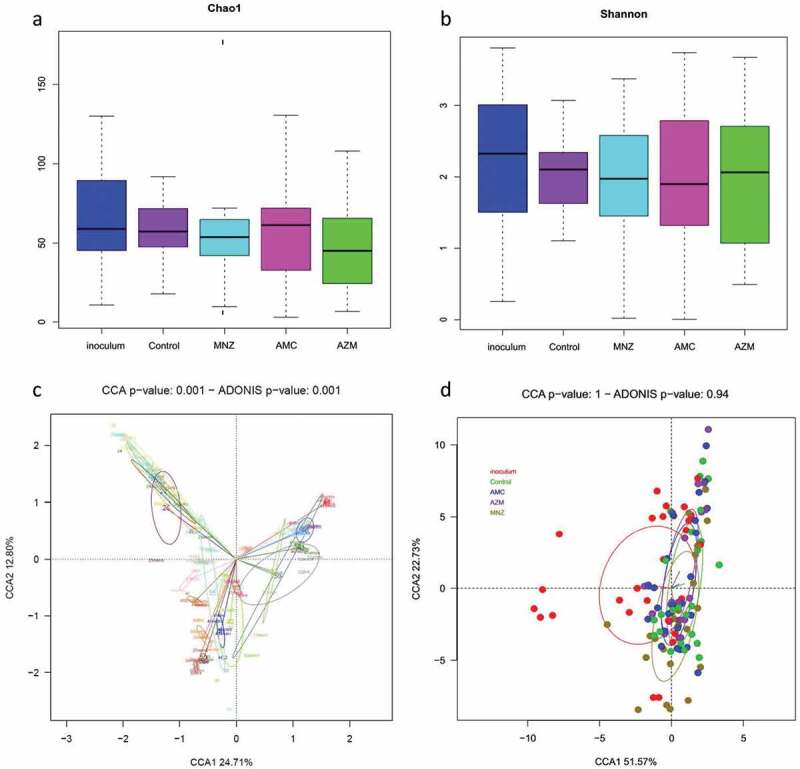
Microbial composition analyses of endodontic infections and their corresponding biofilms grown in the absence and presence of antibiotics. The analysis was performed on all patient samples from which endodontic biofilm grown was obtained in the absence of antibiotics (control) and after the application of amoxicillin + clavulanic acid (AMC), metronidazole (MNZ) and azithromycin (AZM). a) Bacterial richness (estimated number of bacterial species) as determined by the Chao1 index. c) Bacterial diversity as determined by the Shannon index. Lower panels represent a graphical representation by canonical correspondence analysis (CCA) of the microbial community composition of the biofilm as grouped by patient (c) and by antibiotic treatment (d). In panel (c), each circle and color corresponds to a different individual. Each sample occupies a position in the 2-D space according to its bacterial composition at the species level as determined by 16S rRNA sequencing.

To evaluate the effect of the different antibiotics on the biofilm bacterial composition, a canonical correspondence analysis (CCA) was performed. Figure 7c shows that the bacterial composition at the species level varied among the 19 patients included in the analysis (p = 0.001). Furthermore, all the samples from the same patient, after application of the different antibiotics, remained very similar in composition; this indicates that, although some genera may vary in their relative percentages, the antibiotics tested do not influence the overall structure of the bacterial populations. Likewise, when grouping the 19 samples by the antibiotic applied, there was no statistically significant difference between the bacterial composition of the samples treated with the different antibiotics and those of the untreated biofilm (Figure 7d No significant differences in bacterial richness and diversity were observed between the control and the antibiotic treatments, as indicated by their Chao1 and Shannon indexes (Figures 7a and 7b).

In addition, after treatment with a given antibiotic, the bacterial composition did not vary between samples where the biofilm mass increased, decreased or remained the same (Adonis p-value were 0.35, 0.75 and 1 for azithromycin, amoxicillin and metronidazole, respectively) indicating that the antibiotics had a similar effect on most bacteria within the biofilm. However, although the overall bacterial community composition overlapped between control and the different antibiotic treatments, a few bacterial species were significantly affected (ANCOM-BC compositional analysis). Specifically, *Streptococcus cristatus*, *Veillonella parvula*, an unclassified species of *Streptococcus* and an unclassified *Neisseria* were all over-represented after treatment with metronidazole. On the other hand, when azithromycin was added to the media, the abundance of *Staphylococcus* was reduced considerably from 7,5% to 0,7% and *Parvimonas micra* was increased from 0,4% to 1,5%. Finally, the addition of amoxicillin reduced the levels of an unclassified *Lactobacillus* whereas increased the levels of an unclassified species of *Fusobacterium* and *Dialister invisus*.

**Figure 8.**
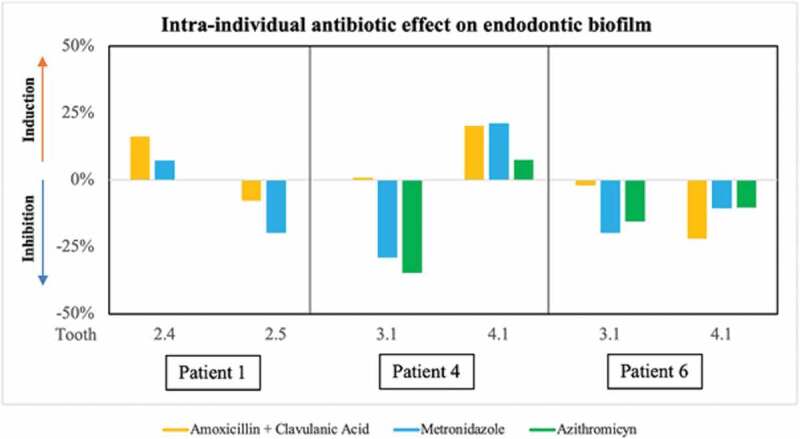
Graphical representation of the effect of the antibiotics tested *ex vivo* on biofilm formation from two samples obtained from each of 3 patients. Data are shown from patients 1, 4 and 6, shown as percentage of inhibition or induction of biofilm formation relative to the control sample without antibiotics at 8 h of growth. The bars show the mean of two replicates.

In three of the patients included in the study, endodontic infection samples could be obtained from two different teeth. Figure 8 represents the degree of inhibition or induction of the different antibiotics on biofilm formation from samples obtained from each tooth of the same patient, showing a different effect. For example, in Patient 1, amoxicillin + clavulanic acid and metronidazole induced biofilm growth in the sample from tooth 2.4, while both antibiotics tested on the biofilm from the adjacent tooth (2.5) inhibited biofilm formation. Similarly, in Patient 4, the antibiotics tested had a different effect on biofilm formation in samples from adjacent teeth (3.1 and 4.1). In contrast, in Patient 6 both metronidazole and azithromycin produced a similar inhibition of biofilm formation in both samples (3.1 and 4.1), while amoxicillin + clavulanic acid in tooth sample 3.1 had no detectable effect, but produced an inhibition of 22% in tooth 4.1. None of these teeth had periodontal disease. In patient 1, the cause of treatment was caries, while patients 4 and 6 had a history of trauma.

### Correlation between clinical and microbiological data

We have tested the possible correlations between clinical parameters and bacterial community composition or antibiotic susceptibility in 40 patients. Specifically, we tested the correlation with spontaneous pain (present in n = 5, representing 12.5% of the patients), dental caries (present in 27.5% of the patients), periapical area (67.5% of the patients), trauma history (20% of patients), sinus tract (7.5% of patients) and percussion pain (22.5% of patients).

The influence of different clinical situations on the antibiotic effect in endodontic biofilm growth was analyzed using the chi-square test. Percussion was negative in 55.6% of the cases in which amoxicillin-clavulanic acid induced biofilm growth, and negative in 40.6% of the cases in which it inhibited biofilm formation (p = 0.03). In 56.5% of cases where no suppuration was found, azithromycin reduced biofilm formation, while it did not affect biofilm formation in any samples with suppuration (p = 0.02). In 44.4% of the cases in which there was an apical radiolucent area, amoxicillin-clavulanic acid inhibited biofilm growth, while in 42.9% of the samples in which there was no area, it stimulated biofilm growth, but the difference was not statistically significant (p = 0.06). No other association was found between the different clinical data collected and the influence of antibiotics on biofilm formation.

Genus and species-level compositions (normalized by ANCOM-BC) were used to analyze the relationship between the clinical data of the patients and bacterial abundance for microorganisms with a proportion of more than 1% in the original sample. A higher abundance of *Prevotella* and *Treponema* were observed when pulp necrosis was caused by trauma, and a higher abundance of *Streptococcus* when caries caused pulp necrosis, but these differences were not statistically significant when corrected for multiple comparisons. The same lack of statistical significance after correction for multiple comparisons was observed for the higher abundance of *Streptococcus* in the presence of pain prior to root canal treatment, and, at the species level, for the lower numbers of *Corynebacterium matruchotii* and an unassigned *Veillonella* species when a radiolucent area was present. Finally, a Canonical Correspondence Analysis between the bacterial composition and the four types of biofilm formation pattern (type I-IV) showed no significance, indicating that the biofilm antibiotic susceptibility pattern cannot be explained by bacterial community composition alone.

## Discussion

It is known that the bacteria causing apical periodontitis, present inside the canals, are organized in complex structures giving rise to a biofilm [37–39]. Interactions between microorganisms in a biofilm endow the community with properties and functions that cannot be observed in each component individually [6]. Thus, an increase in virulence an increased resistance to different antimicrobials and an improved metabolic efficiency have been observed [5]. Therefore, disinfection strategies should be tested under conditions as close to reality as possible.

The microbial composition of the biofilm in the periapical area is similar to that found inside root canals or in sinus tracts, which justifies the use of biofilm samples from root canals in the present study [39,40]. Specifically, an *in vitro* real-time study model was developed for samples from necrotic root canals, and the *ex vivo* effect of four different antibiotics on the growth and bacterial composition of the resulting endodontic biofilm was tested.

Unlike most endodontic biofilm growth systems, in which the species to be tested are artificially selected, in the current study the sample was directly collected from inside the canals. In this way, the results achieved by this ‘microcosm’ system resemble reality more closely, allowing interactions between the different colonies present in the intra-radicular biofilm [8]. The impedance RTCA system developed here, unlike other study models such as the AAA (ACTA active attachment) model [11] or the MAM (multiplaque artificial mouth) [13,41] which are ‘end point’ systems, allows real-time monitoring during the entire period of biofilm formation. This *in vitro* model has been validated for other types of oral samples [15], but the present work is the first to study the dynamics of endodontic biofilm formation, which has mainly been studied in single-species biofilms, primarily with *Enterococcus faecalis* [9,11]. Our data emphasize the enormous diversity in bacterial composition between samples from different individuals, and even between samples from the same individual when more than one tooth was sampled (see Additional Material 1 for individual data). It is important to underline that *E. faecalis*, an organism commonly used as representative of endodontic infections, was only detected in 5 out of 40 samples in the current study, and only in one of them at a proportion >1%. This indicates that more complex and representative *in vitro* models of endodontic infections are required. In relation to this, we show that the initial sample (inoculum) from which the biofilm is formed, and the resulting biofilm are similar in composition after 8 hours of incubation. However, there are slight differences, such as an increase of *Streptococcus mutans* or a decrease in proteolytic organisms like *Fusobacterium* in the biofilm obtained. An increase in Firmicutes is common in microcosm experiments because the presence of sugars in the culture media often stimulates the growth of fermentative organisms [41]. Therefore, endodontic biofilm growth conditions could probably be refined in the future by modifying the culture medium used with reduced sugar levels. In addition, the viability of some strict anaerobes could be compromised from sampling to biofilm growth, and we therefore propose the use of transport media with reducing agents in the future for maximizing viability of anaerobes. Other weakness of the impedance system is that it only studies the initial phases of biofilm formation (<24 h). Thus, although a stationary phase in biofilm growth is obtained after 8 hours in our experimental system, this does not mean that further growth or changes in bacterial composition or activity cannot be produced after that period, especially if fresh medium would be added. It must also be considered that impedance measures can be influenced by ionic components of the culture media and by some antibiotics and therefore appropriate controls are required. It is also important to keep in mind that the system measures biofilm growth indirectly through impedance, although available data support that impedance correlates with total biofilm mass [42] and with other methods like crystal violet staining or CFU counts [16]. Impedance in the current manuscript was selected because of its low-cost, because samples do not need to be manipulated or altered in any way and because available literature supports that the biofilm formed is representative of the complex microbial community inhabiting different oral niches. However, other methods, like real-time imaging [43], could provide a more direct assessment of biofilm growth and should be explored in the future.

The use of dentine discs in an *in vitro* study model would be the substrate closest to reality [8], but this could only be used in ‘end point’ study models. In the current work, a nutrient-rich culture medium (BHI) supplemented with hemin menadione and vitamin K under anaerobic conditions has been used to allow for the growth of as many bacterial species as possible [23]. The incubation time of biofilm usually ranges in the literature from 1 to 70 days [8,11,15,41]. A limitation of the present study is that the biofilm was allowed to mature only up to 8 hours, at which time the stationary phase was reached. Therefore, the effect of antibiotics on mature biofilms has not been tested. However, the assessment of the antibiotic effect at the beginning of biofilm formation is equivalent to the situation of biofilm growth after chemical-mechanical disinfection of the inside of the ducts, which may represent a useful information to know the potential effect of an antimicrobial treatment.

Amoxicillin + clavulanic acid was selected as one of the antibiotics to be tested because amoxicillin is the antibiotic of first choice recommended by different professional associations, including the ESE [17,18], the ADA [44] and the AAE [45] against infections of endodontic origin. It is also the antibiotic most widely used in dentistry according to the available literature. Metronidazole and azithromycin were also used in the present study because, together with clindamycin, they are the most widely used antibiotics after amoxicillin [46–48]. The ADA recommends administering metronidazole to a patient who has no improvement in symptomatology after 2–3 days of antibiotic regimen with amoxicillin; for patients with amoxicillin allergy, it recommends administering azithromycin or clindamycin [44]. The recommended doses are 500 mg for metronidazole and 500 mg for azithromycin [18,46]. Therefore, the present study has evaluated the *ex vivo* efficacy of these widely used antibiotics against endodontic biofilms.

Mira *et al*. (2019) [15] described the real-time formation dynamics of biofilm formed from inocula taken from saliva, tongue scraping and dental plaque using the same RTCA impedance system. In the present study, we have succeeded for the first time in studying the dynamics of endodontic biofilm formation and its susceptibility to amoxicillin + clavulanic acid, metronidazole and azithromycin. The main conclusion from our data is the enormous variability in the response patterns of endodontic biofilms to the antibiotics tested. This large variability occurred even in the three patients from whom 2 different tooth samples were obtained, showing that, even in the same individual, the antibiotic sensitivity is variable and difficult to predict. In the future, it could also be interesting to study bacterial composition in those samples where the antibiotic treatment had no effect on growth, which were not sequenced in the current work.

Most of the antibiotic effect was found to range between 25% inhibition and 25% induction of biofilm growth. Metronidazole inhibited biofilm formation to the greatest extent (>50%), and azithromycin inhibited biofilm formation in the highest percentage of cases. In addition, all four antibiotics have led to biofilm induction in some cases. These results differ from those obtained by Brescó *et al*. (2006) [49], where different antibiotics were applied to bacterial strains isolated from periapical lesions and pericoronaritis. They observed that amoxicillin and amoxicillin + clavulanic acid were the antibiotics with the highest number of susceptible strains, with a statistically significant difference between the number of susceptible and resistant strains. In contrast, no significant difference was found with azithromycin and metronidazole, with metronidazole being the antibiotic with the highest number of resistant strains. These differences in the effect of antibiotics may be due to the fact that bacteria behave differently in pure cultures compared to when they are structured within a biofilm [6]. For this reason, the data presented in this study support the use of real, natural samples to test antibiotic susceptibility, as the behavior of a biofilm with a complex composition -where a multitude of interactions and synergies exist- cannot be inferred from the antibiotic sensitivity of some of the biofilm components in single cultures. In relation to that, the fact that only small changes were observed in the bacterial composition of the biofilms after antibiotic treatment does not imply that the bacteria that form the biofilm are individually resistant to the antibiotic. In fact, it is very likely that if each of the species were tested individually, most would probably be found to be sensitive. The possible explanation for this resistance lies on one hand, in the complex interactions that occur among the bacteria that make up this multispecies biofilm, and on the other hand, the presence of the biofilm matrix that totally changes the individual properties of the bacteria in terms of their individual resistance [6].

Under the tested conditions of temperature and culture medium, there is little difference in the values of inhibition and stimulation of biofilm formation by the tested antibiotics between 4 and 8 hours of monitoring. This means that at 4 hours, preliminary values available are very similar to those that will be obtained at 8 hours. Therefore, the RTCA impedance system is proposed as a rapid *in vitro* model for antibiotic sensitivity testing. It is worth noting that antibiotic sensitivity tests routinely used in the hospital setting, such as microdilution tests or E-tests performed on pure cultures, take at least 24 h to produce a result; in addition, genetic antibiotic sensitivity tests performed on periodontal samples also require at least 24 h for diagnosis [50]. On the other hand, the system presented here requires fresh samples to be cultured within 24 h for optimal results. This could present logistical problems, which could be partially solved by using means of transport for anaerobic organisms [51].

## Conclusions

Our data indicate that *ex vivo* antibiotic treatment has little efficacy against endodontic biofilms and this efficacy is virtually impossible to predict, with many cases where the antibiotic in fact stimulates biofilm growth. For example, metronidazole was the antibiotic with the largest inhibitory effect and azithromycin the one inhibiting in the highest percentage of cases, but both induced biofilm growth in some samples, making it impossible to determine a default preferred antibiotic. Thus, the dramatic sample-dependent pattern revealed by the present study would recommend individualized rapid tests to make a treatment choice that can be more effective while promoting a responsible use of antimicrobials. Furthermore, we propose that a rapid impedance monitoring system on real samples (instead of monospecific cultures) can be a very helpful tool in finding and testing new therapeutic alternatives for endodontic infections.

## Supplementary Material

Supplemental Material
