# Supplementary figures and images for: Ex vivo evaluation of antibiotic sensitivity in samples from endodontic infections
Source: J Oral Microbiol. 2022 Dec 22;15(1):2160536. doi: 10.1080/20002297.2022.2160536 (PMC9793940; doi:10.1080/20002297.2022.2160536)

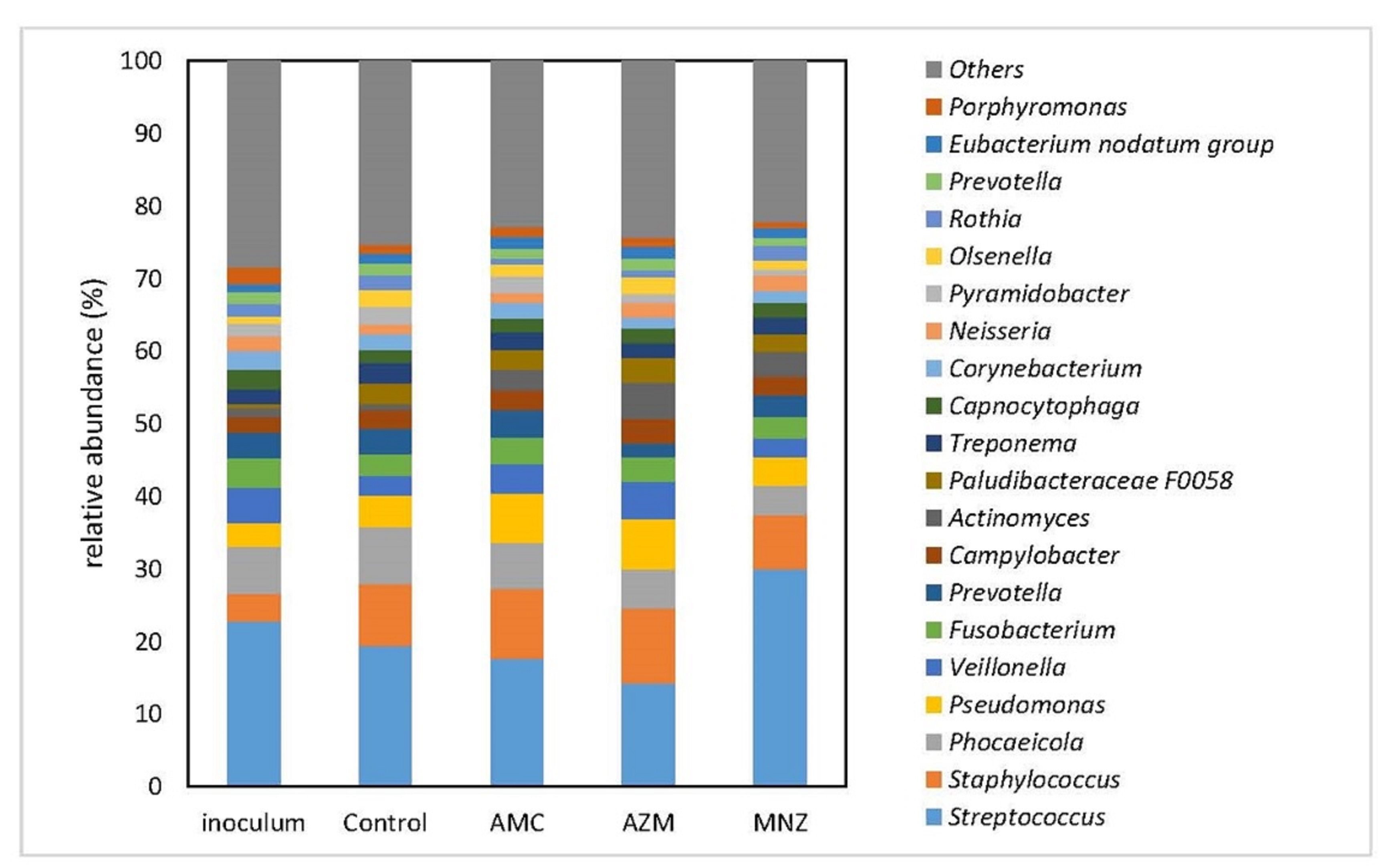

Supplement: Supplemental Material [file ZJOM_A_2160536_SM2910.jpg]
